# A Framework for Identification, Assessment and Prioritization of Climate Change Adaptation Measures for Roads and Railways

**DOI:** 10.3390/ijerph182312314

**Published:** 2021-11-23

**Authors:** Yvonne Andersson-Sköld, Lina Nordin, Erik Nyberg, Mikael Johannesson

**Affiliations:** 1 Swedish National Road and Transport Research Institute, VTI, SE-581 95 Linköping, Sweden; lina.nordin@vti.se (L.N.); erik.nyberg@vti.se (E.N.); mikael.johannesson@vti.se (M.J.); 2 Geology and Geotechnics, Department of Architecture and Civil Engineering, Chalmers University of Technology, SE-412 96 Gothenburg, Sweden

**Keywords:** adaptation measure sustainability assessment, stepwise methodology, cause-effect-relationship

## Abstract

Severe accidents and high costs associated with weather-related events already occur in today’s climate. Unless preventive measures are taken, the costs are expected to increase in future due to ongoing climate change. However, the risk reduction measures are costly as well and may result in unwanted impacts. Therefore, it is important to identify, assess and prioritize which measures are necessary to undertake, as well as where and when these are to be undertaken. To be able to make such evaluations, robust (scientifically based), transparent and systematic assessments and valuations are required. This article describes a framework to assess the cause-and-effect relationships and how to estimate the costs and benefits as a basis to assess and prioritize measures for climate adaptation of roads and railways. The framework includes hazard identification, risk analysis and risk assessment, identification, monetary and non-monetary evaluation of possible risk reduction measures and a step regarding distribution-, goal- and sensitivity analyses. The results from applying the framework shall be used to prioritize among potential risk reduction measures as well as when to undertake them.

## 1. Introduction

Roads and railways are sensitive to climate-related events such as intense rainfall and flooding causing landslides, damaged bridge foundations, overloaded track drainage, flooded tracks and derailment risks, already resulting in high costs [1,2,3,4]. The costs are expected to increase as extreme weather events will become more frequent and severe due to climate change [5]. Moreover, solar radiation, precipitation, and winds constantly wear the road surfaces, railway rails and banks [6]. Climate change might cause such deterioration to increase. In addition, heat waves will affect both roads and railways. While solar curves can be disastrous for the railways, heat waves might cause changes in the interior of the road causing migration of liquid asphalt or bleeding, and ravelling (loss of stones) on the road surface [7,8]. Further, changes in water content, repeated freezing and thawing, moisture content and more severe cloudbursts, high winds, and increased subsurface water will result in increased progressive shear failure, excessive plastic deformation, attrition with mud pumping, frost action, swelling and shrinkage, washout, and slope erosion [5,9]. Enriquez-de-Salamanca [10] showed that an increase in summer temperatures in some regions might increase pavement damage, while higher winter temperatures instead might reduce damages. However, in cold climate regions, an increase in temperature might cause an increase in freeze and thaw cycles, hence, causing more damage to the roads.

To maintain resilient roads and railways it is important to start planning for climate change adaptation measures. Accordingly, several European countries have set up national strategies or roadmaps, e.g., PreventionWeb [11]. However, in the transport sector there is still a need for further investigations, assessments, and methodologies for prioritization [12]. Though, there are some studies that have developed methods for climate risk assessments and analysis of the road and railway system. For example, Chinowsky et al. [13] estimated adaptation costs for the US road network with regard to rutting caused by precipitation, freezing and thawing cycles, high temperature induced pavement cracking, and erosion caused by precipitation on unpaved roads. In the UK, the Rail Safety and Standards Board developed a government vision and policy on adapting infrastructure to climate change [14]. It includes some climate impacts that might affect road and rail as well as suggestions on measures, but not on how to assess risks or prioritize measures. Other studies focus on parts of the problem, either by only investigating the turnouts of the railways system as in Dindar et al. [9], or only focusing on one climate parameter, such as flooding [15]. Bubeck et al. [15] presented flood risk assessments for European railway tracks based on publicly available data about the rails. Their study described flood risks based on several models, but, as it aimed at providing an overview of future flood risks in Europe, it is still very general. In addition to climate hazard assessments, there are studies on risks, costs, and socioeconomic costs related to the road or rail infrastructure (e.g., [2,16,17]). There are also studies that have investigated and suggested methods on how to detect and manage risks and prioritize adaptation measures. Such methods usually follow the same procedure: first a review of which regional climate change factors that are expected, how this might impact the infrastructure, and what kind of adaption measures that should be prioritized—often based on risk or vulnerability assessments [2,12,18].

To understand local risks, vulnerabilities, and potential solutions associated with roads or railways, some studies have collected information from experts via surveys, interviews, or workshops (e.g., [12,19,20]). For example, Bles et al. [20] developed a method, focusing on the TEN-T road network in Europe, that partly builds on workshops. A common understanding of the risks, investment and running costs, as well as socioeconomic costs are beneficial for a smooth, effective, and well-informed decision process. For complex infrastructure projects, with high degrees of uncertainty, such a process shall be based on collaboration, explorative learning, and adaptation [21]. However, as illustrated by a case study in Norway, it may be difficult to encourage public authorities to work together in understanding the socioeconomic effects of, for instance, a road closure in an avalanche risk area [22]. It is therefore important, and challenging, to develop methods for climate adaptation that are easily comprehended and at the same time include a well investigated background regarding local risks and vulnerabilities, as this makes it easier for nonexperts in municipalities to understand and accept the risks, as well as act on them [23]. In addition to socioeconomic impacts, adaption measures may result in positive environmental synergies as well as unwanted environmental impacts. Accordingly, various studies have investigated the environmental gain of planning for adaptation measures that could also mitigate climate change [10,13,19,24,25,26]. Despite the existence of several methods to either assess risks, or the costs related to adaption measures, there is a lack of methods facilitating well-informed decisions among authorities or operators on risk and prioritization of adaption measures.

There are, to our knowledge, currently no methods which include all of the necessary steps, from hazard identification to the final steps of assessing the risk reduction, and the related costs and benefits, from a sustainability perspective, including which measure to undertake when (e.g., [27]). There is also a need for decisions to be taken based on current knowledge and available data, as well as development towards better and more covering data, and a better understanding of the full cause-effect-relationship. This article presents a framework to assess the full cause-effect-chain from the identification of hazards to risk assessment and valuation of risk reduction measures on road and railway systems. It also presents identified knowledge gaps. The results of applying the framework shall be used to prioritize among risk reduction measures and when to undertake them. The framework is generic, but originally developed for the Swedish rail and road system. To test its applicability, it is used on a flood event in Lödöse, Sweden.

### Climate Adaption in the Swedish Road and Rail Infrastructure

In the Swedish road and rail infrastructure, climate change is expected to cause an increase in annual precipitation (snow and rainfall), average temperature, sea level, and more frequent and extensive extreme weather events. This will result in a significant increase in disruptions and socioeconomic costs that are already expensive. Therefore, in 2017 the Swedish Transport Administration (STA) initiated strategic work on how to adapt the roads and rails to climate change [28]. However, measures can be costly and may result in unwanted consequences. For example, how the infrastructure is dimensioned and designed may impact both the constructions’ technical solutions and functionality, and the landscape. Accordingly, the STA aims to undertake measures that are cost-effective and optimizes the benefits over the costs, i.e., that minimizes the total expected socioeconomic costs of damage and adaptation measures [28,29,30].

To be able to assess the costs and benefits for relevant climate scenarios and various lifetimes of the constructions, reliable impact-relationships are needed in both the planning of new constructions and in adapting the existing ones [28,30]. However, the STA [30] identified that there are several knowledge gaps, a lack of methods covering the full cause-effect-chain, and a great need for more knowledge to be able to undertake the most relevant measures. In response to this, we present a scientifically based conceptual framework that aims to follow the cause-effect-chain, from identifying climate-related risks and measures, to how to assess the costs and benefits of the potential measures to reduce climate-related road and railway risks, in both a short and long-term perspective. The framework aims to be used for assessing climate-related consequences that are important in regards to the STA’s facilities and in assessing the costs and benefits of risk reduction measures, considering where and when to undertake which measure. To investigate its relevance and applicability it was also tested on a case study. The following research questions were addressed: which methods should be applied to be able to assess climate related risks and to prioritize among risk reduction measures; can they be applied in one common framework; is such a framework of relevance for real case applications; and are the available methods and data adequate?

## 2. Materials and Methods

The conceptual framework was developed based on white (academic) literature and reports on, and experiences of, weather-related events such as fires, flooding, landslides etc. at the STA and in Sweden. This information was combined with previously developed methods for cost-effectiveness applied within the STA [31], classic risk management methodology (ISO 31000, [32]), and multi-criteria methodology (e.g., [19,33]). Moreover, the available methods for risk assessments of the individual climate-related risks have been used, e.g., methods for assessing risks related to heatwaves and methods for assessing flood-risk, and the dewatering capacity, erosion, and landslides. The methods have been found in academic literature as well as webpages (e.g., the STA, PreventionWeb). In addition, interviews were conducted to achieve knowledge on the experiences of climate-related risks, their trends, and the current management strategies for those within the STA. For this, interviews were conducted with experts as well as personnel working with operation and maintenance daily. The interviewees were selected by identifying experts via Swedish webpages and by using the snow-ball principle. The respondents were initially contacted via email. The aim was to identify their interest, ensure that they were willing to take part in the interviews voluntarily, and to allow their responses public but neutralised (i.e., freely and actively given consent). If so, they were also asked to suggest time for an interview. The interviews were conducted using Skype. Further, all interviewees were asked if they preferred being anonymous (which no one wanted) and if so, how to be referred to in text. They also had the opportunity to read, correct and take part of the results of the work.

The aim with the interviews was to cover current and previous experience and expertise in relevant fields, e.g., one of the interviewees was the specialists on rail and road and was responsible for climate change related aspects in the Swedish Commission on Climate and Vulnerability [34]. The other interviewees were national experts at the STA on water management or responsible for planning, operation, and maintenance at different levels within the STA (national planners and managers, regional managers, and those in daily contact with contractors). Additionally, experts on climate risk and adaption experiences in other organisations (SGI, VTI, Chalmers University of Technology, Gothenburg, Sweden) were included among the interviewees. The identification of new interviewees ended when the same names as already interviewed were provided, and when the replies among the interviewees were similar to previous interviews. In total, 14 interviews were conducted, and the resulting interviewees are presented in Table S1 in the Supporting material. The interviews were qualitative and not aimed at achieving quantitative information.

The interviews were conducted in order to identify the needs and current strategies within the STA. The results of the interviews were used as the starting point of the development of the conceptual framework. Furthermore, the development of the conceptual model was conducted in cooperation with the funder of the project, who at that time was responsible for climate adaption at the STA, and an economist involved in the climate adaption strategy developments at the STA. They also contributed with experiences from the internal discussions and ongoing work on climate adaptation within the STA.

**Figure 1:**
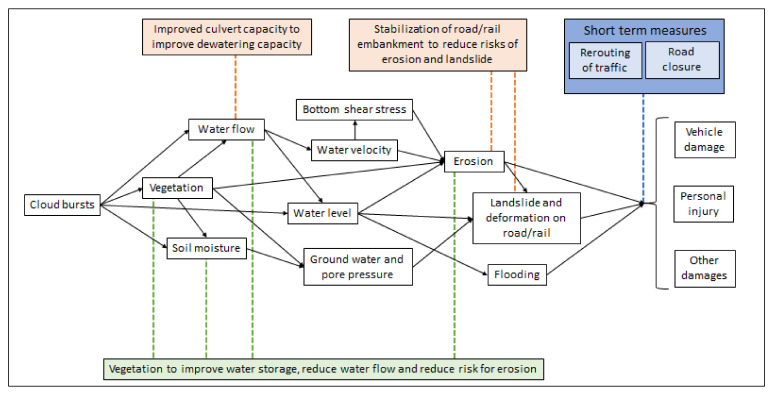
Potential impacts of a cloudburst, and examples of preventive (long-term, brown and green text boxes) and more acute (short term, blue text boxes) measures and where they will impact the cause-effect chain.

A cause-effect-relationship for climate adaptation measures implies that a defined measure is expected to provide a certain effect, i.e., a certain reduced risk. The risk is a combination of consequence and the likelihood (probability) of this consequence to occur. The consequence is the impact of the climate-related event on, for example, accessibility, injuries and fatalities, and damages on vehicles and installations. The consequence can be valued monetarily by calculating an expected cost of damage. The probability of each consequence needs to be assessed through several steps since climate-related events, such as a heavy rainfall, may cause several impacts, e.g., flooding, erosion, and landslides, as illustrated in the example with a cloudburst in Figure 1. Each of these consequences may in turn lead to further consequences such as damage to a road or rail, which in turn may result in accidents, fatalities, or vehicle damages. If the road damage is severe, it may require the road to be closed for a rather long-time during reparation. When closing a road, traffic needs to be diverted, which in turn can lead to an increased probability of a road accident. Therefore, in this paper risk is used to describe the combination of the probability of a specific climate-related event, such as the likelihood of heavy rainfall with a certain return time, as well as the consequences that may arise from this climate-related event. To assess the probability of climate-related events, return times based on statistics on past weather events, e.g., heavy rainfall, are used. When available, site specific or local scale data shall be applied (e.g., [35]). To provide probabilities for future events, the statistically based return times are combined with projections of future expected climate change. As a basis for assessing the short- and long-term climate related consequences for the Swedish Road and Rail infrastructure, regional climate scenarios [36] and previous national and international assessments of potential climate change impacts in the transport sector can be used.

A risk reduction measure can be reactive or proactive. A proactive risk mitigation measure can be preventive in reducing the likelihood of an event, e.g., the likelihood of a certain flood depth, the consequences of the event, or both simultaneously. Measures may vary depending on the risk it aims to reduce. As illustrated in Figure 1, a measure such as larger road drums will reduce the likelihood of flooding and any further impacts, while a measure such as stabilizing rail embankment and road banks can reduce the likelihood of erosion and landslides (and its further effects), but not the likelihood of flooding. A measure, such as a well-designed vegetative solution, may provide risk reduction at more than one stage of the impact-chain (Figure 1). Often, a preventive measure taken earlier in the chain of impact is more cost-effective than a measure taken later in the chain [37,38,39]. To assess the costs and benefits of a risk reduction measure, the effect should be verified, i.e., based on science or proven experience. Further, the effect estimates need to be site-specific. The estimated effects can be valued and assessed in monetary terms or by other quantities, or in relation to other effects. In addition to the damage costs, the total cost includes the sum of delay and road closure costs and may include, e.g., diversion and interchange costs. Injuries, fatalities, and environmental damages, as well as material damage beyond transport infrastructure, are also included. There may, for example, be an increased risk of accidents during traffic diversion. The cost in terms of transport and accessibility is affected by many factors, including: suspension time, traffic intensity based on AADT (annual average daily traffic), type of passenger and freight modes, changes in travel times, opportunities for diversion, and access to alternative modes of transport. The transport related costs can be calculated with existing models, such as the traffic and transport models available at the STA [40], and the travel time values available in e.g., ASEK 7.0 (ASEK is a Swedish abbreviation meaning the principles and values that are recommended to be used in social cost-benefit analyses (CBA) in the Swedish transport sector) [41]. To assess the applicability of the resulting framework, it was applied on a case study, Lödöse, in Southwest Sweden.

## 3. Results

### 3.1. The Framework

The conceptual framework consists of four steps with major questions to be answered. The answers shall be used to assess whether it is socioeconomically relevant to implement a measure, when it should be implemented, and to assess which measure is most relevant to implement. The process includes the following steps:

- Hazard/risk identification for climate related events;
- Risk analysis, i.e., assessment of probability, consequences, and valuation of the risk
- Identification and evaluation of possible measures, including cost benefit analysis and ranking of measures;
- Distribution analysis, goal analysis, and sensitivity analysis.

The major steps and questions to be answered through the process are based on classic risk management [32] (steps 1–3) and a method developed at STA for impact evaluation [31,42,43] (steps 3 and 4). Even if the steps are consecutive, the process of applying the framework should be iterative.

#### 3.1.1. Step 1. Hazard Identification

In the hazard identification there are two basic questions to be answered:

- What climate-related changes can be expected in the short (current climate), medium, up to year 2040, and long, up to year 2100 (or longer depending on the construction lifetime), term perspectives?
- What consequences can these changes be expected to cause within the STA’s areas of responsibility and activity, and which of these consequences are important to take further into account?

The climate change-related weather events and climatic changes that are relevant to consider can be identified based on literature, webpages and interviews, and climate scenarios [36,44]. The aspects to consider include temperature; (temperature) zero crossings (when the highest temperature of the day two meters above the ground has been above 0 °C and the lowest temperature in the same day has been below 0 °C) and frost; precipitation, type of precipitation (rain, snow) and precipitation patterns; sea level; and winds (speed and directions). The hazard and consequence identification can, as here, be based on previous experience (interviews), reported events and experiences, databases, previous investigations, field investigations and expert judgements [18,32,45]. Table 1 shows a summary of climate change related weather events and their consequences that have been identified as important to consider in the transport infrastructure sector.

#### 3.1.2. Step 2. Risk Analysis and Risk Assessment

In this step the following two questions shall be answered:

- How likely is the event (e.g., a torrential rain causing a flood of a certain depth) and what would the cost of damage be?
- Is the risk and cost acceptable?

Risk is a function of probability and consequence. To assess the risk the probability, P, of a particular climate-related event occurring during a given period, the type and magnitude of consequence must be estimated. The consequence can be valued monetarily by calculating the expected cost of damage for a certain period, or estimated on a relative scale (no, low, medium, high, catastrophic etc.). Often, the risk is illustrated by a risk matrix (see example in Figure S1).

The probability is described by the return time. The return time of an event means that the event, on average, occurs or is surpassed once during this time. Today’s return times are based on local/county level statistical data on mean and extremes temperature, precipitation, wind, water tables, sea level, and water flow (e.g., for Sweden, such data can be achieved from SMHI [57] complemented with data achieved from other national and local authorities). To assess the probability in a medium- and long-term perspective, the expected changes (frequencies and amplitudes) due to climate change and future return times are based on results from simulations with climate models and, when not available, observed trends. For example, since the 1950s, heat extremes (including heatwaves) have become more frequent and more intense, and the frequency and intensity of heavy precipitation has increased over most land areas for which observational data are sufficient for trend analysis [5]. In the US, over the period from 1910 to 2020, the portion of the country experiencing extreme single-day precipitation events increased at a rate of about half a percentage point per decade [46]. The prevalence of extreme single-day precipitation events has increased substantially since the 1980s, in comparison to the period 1910 to 1980 when it remained fairly steady, and 9 of the top 10 years for extreme one-day precipitation events have occurred since 1996 [58]. Unless local or regional projections are available, the estimated probability in a medium- and long-term perspective is achieved by adding the expected annual average change to the current return times. The consequence assessment shall be based on observed consequences from previous events (e.g., from databases and event reports), as well as ex-ante modelling, field investigations, and stakeholder and expert judgements [19,33,45]. Table 1 provides examples of methods and data (including relevant references) that can be applied to make ex-ante risk estimates of climate change related weather events.

The damage cost is the sum of repair costs, delay, and closure costs, and may also include diversion costs, interchange costs, and recovery costs. In addition, injuries and direct damage to vehicles may also occur as a direct consequence of the event, for example a landslide, or indirectly, for example by increasing the accidental risk during traffic diversion. The damage cost in terms of travel time and accessibility depends on road closure time, traffic intensity (based on AADT), the type of traffic impacted (passenger/goods), traffic diversion, and access to alternative modes of transport. This can be calculated with traffic and transport models, such as those available at the STA [40], and the travel time values, such as those in ASEK 7.0 [41]. At the STA, there are also standardized values for delays caused by a speed reduction [59]. Due to, for example, subsequential effects on other trains or vehicles, cracked timetables, etc., the delays can be much greater than the initial speed reduction. The extent of the subsequential effects depend on how much of a road is affected, the capacity utilization by rail, the diversion capacity, and if it is single/double track, alternative routes, etc. There is therefore a great need for research and investigation to better predict the extent and duration of the subsequential effects of delays and the cost that can be related to the access to socially important activities affected by delays and cancelled traffic. Closure time, and need for diversion and alternative transport, depend on the type of climate-related event and its impact on the road/rail construction. The risk assessment shall be based on current climate and expected climate change over 20 years, and for longer-life constructions, expected changes up to the construction’s economic lifetime. The risk assessment shall include planned changes, such as investments in infrastructure, and other expected changes, such as changes in traffic flows, land use change, etc.

In classical risk management processes, to answer the question of whether the risk can be regarded as acceptable, criteria are needed for what can be considered as acceptable risk, i.e., the balanced function of probability and consistency. Tentatively, for an initial assessment, a risk matrix divided into five probability classes (from extremely low to a tangible probability of an event occurring) and five consequence classes (from very little unwanted consequence to catastrophic consequence) can be used [18,60], as illustrated in Supplementary Materials Figure S1. If the risk is deemed acceptable, no additional measures need to be taken regarding the specific events, nor is further analysis regarding the mitigation measures for climate-related events. However, it may be appropriate to carry out continued analysis if a measure is justified for other reasons. In some cases, an action that has several positive effects in other areas as well could be justified to implement, even if the measure management of climate-related effects cannot justify its implementation. For those risks that are not deemed acceptable, a further analysis as set out below is needed. In the further analysis, even if the risk initially was deemed as not being acceptable, the measures may be considered too expensive in relation to the benefits they bring. In these cases, the option of not carrying out an action may be the most socioeconomically relevant.

Risk management is often connected with knowledge sharing and lessons learned. The latter is one disaster risk management path for building a resilient society. Knowledge sharing and learning in a collective context (organizations) may be successful ways of addressing some of the problems posed by natural hazards [45], as in the management of complex infrastructure projects in general [21]. The process of using previous experience is of great importance. For example, the flood events in Central and Eastern Europe in the beginning of the twenty-first century led to preventive actions [61,62,63]. The EU Flood Directive [64] was implemented after severe floods in Europe. The flood directive emphasizes that European countries must incorporate proactive measures in their ongoing efforts to become more resilient [62,63]. Further, the downsides of measures taken for protection should also be highlighted, such as protective plans that have led to level effects of false safety from flooding [65].

#### 3.1.3. Step 3. Identification and Evaluation of Possible Measures

This step includes four questions to be answered:

- What potential risk reduction measures are possible to undertake at all?
- How effective is the potential measures, i.e., how does the measure affect the risk (probability and consequence) of a particular event occurring with a specifically expected return time?
- What is the cost-effectiveness of the measures?
- What are the costs and benefits of the measures?

To assess the feasibility and prioritize the measures, an identification of potential risk reduction measures is required. This can be conducted by identifying what measures have been implemented in previous similar events, as demonstration projects or pilots (e.g., [66,67,68]), or ex-ante through interviews with experts and those working in the areas and with activities that may be impacted by the risk and the potential measures. Measures can range from increased preparedness to increased maintenance, temporary solutions, and measures such as increased dewatering capacity through vegetation or stationary protection to reduce the risk of flooding. Some examples of potential measures are provided in Table 1.

The economic relevance of implementing potential measures can be based on the expected reduced risk i.e., the expected effect of the measure over a certain time, and the net present value, NPV, related to the risk reduction. The NPV includes estimates of the investment costs, changes in operational expenses, as well as the expected changes in risk related costs, in short, medium, and long term. The cost related to an accident depends on several aspects in addition to the damage and repair costs. A large amount of the cost is related to the time of road/rail closure and redirection, in which the cost depends on the AADT and the impacts on the traffic speed and flow. The speed can be reduced due to increase risk related to wheel-tracks and accidents caused by, for example, bleeding asphalt or flooding. The change in speed or the need of closure caused by a flood depends on the water depth and the water flow [69]. The traffic speed needs to be reduced, but the mobility is still possible at 0.2 m depth if the water flow is less than 1.5 m^2^/s. At higher water flows, the mobility is also impacted. Deeper flood depths may also result in reducing the mobility of heavier vehicles, which may challenge the rescue abilities under a flood event [69]. In addition to depth and water flow, the consequence depends on how much traffic is affected (AADT on the current route), how long the flood lasts, and the recovery time required after the event. For example, the duration of a severe flooding linked to temporary sea level rise has so far lasted a few hours in western Sweden. It can therefore be expected to last a few hours up to 24 h also in the future. The time for torrential rain and other heavy rains is also limited. However, the time during which the road and rail traffic is impacted may be significantly longer since measures, such as restoration after damage or the pumping time to get rid of the water, can be much longer. Furthermore, prolonged rainfall, or snowmelt, can cause long lasting flooding. Damage to road or rail, e.g., because of erosion or landslides, can imply that access to the route is completely reduced. The closure time can be assessed based on past events and expert judgements. The consequence, and therefore the cost, as well as any additional consequences, also depend on the diversion and/or alternative transport possibilities and the availability of early warning systems and preparedness.

As risk-mitigating measures can lead to both more positive and more adverse consequences than those related to the risk-reduction itself, a better understanding of what other benefits and costs that may arise from various measures is essential. For example, different types of greenery can be used both to reduce the risks of flooding, erosion, and landslides at the same time as it may contribute to reduce the spread of pollution and increase peoples’ well-being by making a place more attractive. However, greenery can also increase the fire risk or affect other soil infrastructure issues that should be considered accordingly. Various measures also affect greenhouse gas emissions and other pollutants and resource use to different extents. Such impacts should also be considered when prioritizing among measures. However, there is a lack of quantifiable and monetary values for many of the aspects that should be considered in the socioeconomic analysis, which therefore needs to be developed.

To prioritize possible measures, a cost-benefit analysis (CBA) can be used. In CBA, the net present value is the estimated monetary difference between the benefits and the costs over a certain time horizon. Data for the valuation will be relevant international internalizations, or national such as the Swedish ASEK 7.0 [41], that are currently applied for travel time, accidents and fatalities, climate effects, air pollutants, and noise; knowledge and results from previously available estimates on changes in mode of travel, changes in congestion, and parking fees; values of relevant ecosystem services and available meta studies in the literature. Accordingly, NPV can be calculated by Equation (1).

(1) $$NPV=\sum_{t=1}^{k}P*D*{\left(1+r\right)}^{-t}-C_{I}-\sum_{t=1}^{k}C_{M}*{\left(1+r\right)}^{-t}=P*D*\left(\frac{1-{\left(1+r\right)}^{-k}}{r}\right)-C_{I}-C_{M}*\left(\frac{1-{\left(1+r\right)}^{-k}}{r}\right)$$

where

P is the probability of accident,

D is the damage cost of accident,

r is the discount rate,

k is the economic life span of preventive measure,

$C_{I}$ is the investment cost (including the marginal cost of public funds, MCPF), and

$C_{M}$ is the maintaining cost (including the marginal cost of public funds, MCPF).

Incorporating that the probabilities of climate related risks are likely to increase over time in the CBA could be acquired by letting the probability increase exponentially over time:

(2) $$P_{t}=P_{initial}*e^{\frac{\ln\left(\frac{P_{final}}{P_{initial}}\right)}{y}t}$$

where:

$P_{initial}$ is the initial probability,

$P_{final}$ is the final probability, and

y is the number of years the increase in probability is occurring during.

The NPV can thereby be estimated by Equation (3).

(3) $$NPV=\overset{k}{\underset{t=1}{\sum }}P_{t}*D*{\left(1+r\right)}^{-t}-C_{I}-\overset{k}{\underset{t=1}{\sum }}C_{M}*{\left(1+r\right)}^{-t}\;=P_{initial}*D*\left(\frac{e^{\frac{\ln\left(\frac{P_{final}}{P_{initial}}\right)}{y}}}{\left(1+r\right)}\right)\left(\frac{1-{\left(\frac{e^{\frac{\ln\left(\frac{P_{final}}{P_{initial}}\right)}{y}}}{\left(1+r\right)}\right)}^{k}}{1-\frac{e^{\frac{\ln\left(\frac{P_{final}}{P_{initial}}\right)}{y}}}{\left(1+r\right)}}\right)-C_{I}-C_{M}*\left(\frac{1-{\left(1+r\right)}^{-k}}{r}\right)\;=P_{initial}*D*\delta \left(\frac{1-\delta^{k}}{1-\delta }\right)-C_{I}-C_{M}*\left(\frac{1-{\left(1+r\right)}^{-k}}{r}\right)$$

where:

$$\delta =\frac{e^{\frac{\ln\left(\frac{P_{final}}{P_{initial}}\right)}{y}}}{\left(1+r\right)}$$

Building on this, it is possible to calculate the NPV where inaction occurs for a period of time.

(4) $$NPV_{i}=D*\left(-\sum_{t=1}^{i}P_{initial}*e^{\frac{\ln\left(\frac{P_{final}}{P_{initial}}\right)}{y}}*{\left(1+r\right)}^{-t}+\sum_{t=i+1}^{i+k}P_{initial}*e^{\frac{\ln\left(\frac{P_{final}}{P_{initial}}\right)}{y}}*{\left(1+r\right)}^{-t}\right)-C_{I}*\;{\left(1+r\right)}^{-i}-C_{M}*\sum_{t=i+1}^{i+k}{\left(1+r\right)}^{-t}=P_{initial}*D*\delta \left[\delta^{i}\left(\frac{1-\delta^{k}}{1-\delta }\right)-\left(\frac{1-\delta^{i}}{1-\delta }\right)\right]-{\left(1+r\right)}^{-i}\left[C_{I}+C_{M}*\left(\frac{1-{\left(1+r\right)}^{-k}}{r}\right)\right]$$

where:

i is the time of action, $i\in \left[0,y-k\right]$.

Not all costs and benefits are possible to value in monetary terms. Thus, an integrated CBA and multicriteria analysis (MCA) is recommended. Multicriteria methods for prioritizing between potential climate related risk reduction measures are available (e.g., [19,33]).

A more holistic assessment is a combination with a mixed approach where the CBA and non-monetary MCA are integrated. In such an integrated evaluation of costs related to the measure and reduced risk including changes in travel time and accident-related costs and values are combined with evaluation of aspects not monetary valued [69]. Additionally, many of the externalities that are valued in monetary terms (the costs are internalized) can usually not be conducted in an undisputable way. Both the monetary valued effects and other effects of the potential risk reduction measure option should be assessed in relation to a reference (zero) option to be consistent. An overall index (final grade) can be calculated for studied action options *i* (Equation (5)) [70]:

(5) $$H_{i}=100\left[W_{E}\frac{H_{E,i}}{\max\left[Max\left(H_{E,1\ldots N}\right);|\;Min(H_{E,1\ldots N}\right]}+W_{S}\frac{H_{S,i}}{\max\left[Max\left(H_{S,1\ldots N}\right);|\;Min(H_{S,1\ldots N}\right]}\;+W_{NPV}\frac{H_{NPV,i}}{\max\left[Max\left(H_{NPV,1\ldots N}\right);|\;Min(H_{NPV,1\ldots N}\right]}\right]$$

where:

$H_{i}$ is the weighted rating of the alternative risk reduction measure option *i,* where *i*∈[1,N],

$H_{E}$ is the unweighted environmental rating,

$H_{S}$ is the unweighted social rating,

NPV is the net present value, and,

W is the weight of each dimension.

Depending on who participates in the weighting procedure, the importance of the dimensions may vary. Those who carry out the analysis may thus affect the relative importance between the dimensions. From a sustainability perspective, the environmental impacts must set the frame; the (wanted) social impacts are what to strive for; while the aspects included in the economic dimension are the means to achieve a sustainable social and environmental development (e.g., [71]). The basis for those analyses can be literature, questionnaires, in depth interviews and expert assessments by group interviews, and Delphi study approaches. As stated by Eriksson et al. [21], for complex projects there is a need of more flexible project management, including collaboration and explorative learning to smoothen and improve the process. Not least in the weighting process, conflicts of interest must be considered. The process will benefit by including the majority of stakeholders as well as experts on the aspects included in the weighing [19,33].

#### 3.1.4. Step 4. Distribution Analysis, Goal Analysis and Sensitivity Analysis

In accordance with the global Sustainability Development Goals, SDG (Agenda 2030), not only the average costs and benefits are to be considered but also which groups of people that are impacted and to what extent the measures are good for all, or even favoring vulnerable groups. Therefore, a distribution analysis is also needed both to be performed and considered in the evaluation of potential measures. When available and applicable, local, regional, or national methods for distribution analyses are suggested to be applied. For example, the STA has a set of aspects that always shall be considered in traffic projects [72], including how different groups, companies, and regional markets are impacted by the project. To ensure that the measures contribute to the global sustainability goals, those must be included and evaluated in the MCA. Since the SDG are general for a global context, they must be translated to local and county level contexts for the analysis. Currently, there are no standard methods applied for such translation, but it can be based on national and local/county level policies including the same or similar aspects such as the Swedish National Transport goals [73], recommendations and regulations, as well as stakeholder participation methods through interviews and workshops. In both the CBA and MCA assessments, as the final distribution and goal analysis, sensitivity analysis is crucial both to identify which parameters and variables that have most impact on the result and to identify if any of those are as uncertain that additional or deeper assessments are needed for the assessment to be regarded as robust enough. Sensitivity analysis can be conducted by, for example, analysing the impact of applying median, mean, minimum, and maximum values of uncertainties, using Monte Carlo simulations. The sensitivity analysis thereby also contributes to allow a higher degree of uncertainties in the assessments.

### 3.2. Case Study Application of the Framework

The framework was applied to a case study based on a previous flooding that occurred on highway 45 (currently E45) in Lödöse, an urban area located by the Göta River about 40 km northeast of central Gothenburg, Sweden, on 3 December 2011. The road was flooded and the traffic in both directions was stopped for roughly 11 h (13.05–23.59), resulting in socioeconomic consequences due to traffic being stopped, queuing, and rerouting that increased travel distance and time [74]. There is no official information available regarding the cause. In the media, various reasons were provided by different journals (e.g., the flooding was caused by heavy precipitation, flooding in a nearby stream or a further away dam-break).

#### 3.2.1. Step 1. Hazard Identification

Here, the hazard was identified based on the previous flood event at the case study site, and from previous risk estimates on erosion and landslides [75,76], combined with the projected increase in precipitation due to climate change, in the area [36].

The site is in a region classified as an area of vigilance according to Geological Survey of Sweden (SGU) [75]. This implies that the natural conditions are such that a landslide may occur, i.e., the soil consists of clay and/or silt and the ground slope is large enough. These natural conditions allow landslides to occur more or less spontaneously, but not necessarily. The site is in a region with quick clay [76], which is a soil that changes from normal firm ground to a liquid mass when it is disturbed. The liquification is caused by a collapse of the mineral skeleton by shock or other strain and is accompanied by a sudden, temporary increase in the interstitial water pressure and a sudden, large decrease in the shearing resistance. Therefore, consequences in case of erosion and a rather small initial landslide may be large and severe. From previous assessments, erosion and landslide risks in the area are expected to increase with climate change due to increased annual precipitation, more intense precipitation, and increased flows combined with drier summers [37]. Accordingly, landslides with severe consequences may also occur and are therefore considered in the subsequent analysis.

#### 3.2.2. Step 2. Risk Analysis

To estimate the probability of a similar or more severe event under current climatic conditions, previous data from nearby SMHI weather stations [35] was applied. However, the available data from the nearby weather stations did not cover precipitation data applicable to estimate the return time of such precipitation event. Instead, less nearby stations, combined with statistics based on previous larger scale analyses by SMHI [77], were applied to estimate that the event corresponds to a 100-year event.

According to the IPCC, at the global scale, extreme daily precipitation events are projected to intensify by about 7% for each 1 °C of global warming [5]. In Sweden, the annual temperature is projected to increase by more than 4 °C from year 2000 to 2100 [77]. The annual precipitation in the area is projected to increase by up to 40% and the daily precipitation at the day with the most precipitation per year is projected to increase by up to 50% during the period 2000 to 2100. Moreover, the number of days per year with heavy precipitation is projected to increase by up to 20 days in 2020 compared to 3 in 2000. However, the uncertainties are high and there are large variations depending on several factors, such as which climate scenario and regional model that has been used in the simulations [77]. Both the lack of statistical information from nearby weather stations, and the uncertainties in projected frequency and magnitude of heavy rain events in the future, need to be reduced for more certain risk estimates. There are ongoing developments, for example as seen in the latest IPCC report [5], which will provide better estimates for assessing both the current risk and the changed risks by the end of the century due to climate change. Here, based on the compilation of available information, we apply that the probability of a heavy rain event such as the one in Lödöse on 3 December 2011, or heavier precipitation, will increase from a current frequency of 100 years to become a every tenth-year event by 2100.

There is no available information on the annual probability of landslides neither under climate change nor until 2100 beyond the fact that the probability will increase due to the changed precipitation, hydrological patterns, and possibly also due to other changes in the soil such as temperature, fauna, flora, and other biological changes [27]. To be able to predict the impact of those complex ground system changes in quantitative terms as a function of current, as well as a changing, climate, there is an urgent need to achieve an increased knowledge based on field and laboratory studies as well as model development and model simulations. As a sensitivity analysis, calculations were performed changing the probability of flooding, from corresponding to a 1000-year event to a 100-year event (Supplementary Table S6).

Information regarding the current traffic situation of the E45 road, the observed number of hours of road closure and assumptions regarding diversion through the nearest possible redirection routes were used to estimate the socioeconomic costs of the flood event. For this, the STA’s system on current diversion routes, road section lengths, AADT, and speed limits [78] was applied, for details see Table S2 in the Supplementary Materials. The additional distance and travel time of traffic was estimated, per vehicle and in aggregate, and was used to estimate the socioeconomic costs related to the event. Coupled with marginal costs for additional vehicle kilometre and vehicle hour compiled by ASEK 7.0 [41], a socioeconomic cost was estimated for the closure. Since the shadow prices in ASEK 7.0 are mostly divided into four categories; passenger car (PC), passenger car in commercial traffic (PCC), lorry without trailer (L), and lorry with trailer (LT), the same categorisation has also been used here. The shares of PC/(PC+PCC) and L/(L+LT), 90 and 57 percent, are from ASEK 7.0 and the Swedish Road Administration [79], respectively. We assumed that all traffic for the first 5 h was affected by queuing, yielding an average queuing time of 2.5 h for the first 5 h of traffic. While this might be seen as a strong assumption, we also assume that traffic will flow as usual after this time, both on the diversion route and the original road after clearing the road. The resulting marginal costs for additional vehicle kilometre and vehicle hour are summarised in Table S3. Based on the assumed return times, the AADT and the increase in queue time, the socioeconomic cost of a similar event is estimated to 11.2 million SEK in 2017-year prices. A summary of the estimated socioeconomic cost per item is displayed in Table S4.

The closure time related to a landslide affecting the road will be much longer than the closure related to the flood event. From a previous study, based on previous landslide events, transport modelling, and interviews, a landslide at the same location as the flood event was expected to last 33 days and require reconstruction of 100 m of the road [80]. The cost was then estimated to vary between 35–70 MSEK (in 2017-year prices), including restoration of the road (13 MSEK in 2017-year prices) and costs related to the traffic redirection. The cost-variation depends on redirection options including travel time and aspects such as increases in car accidents and impacts on the stability of the redirection roads.

#### 3.2.3. Step 3. Identification and Evaluation of Possible Measures

Three preventive measures to reduce the consequences of similar (or heavier) precipitation event, as well as the probability of a landslide, have been included in the investigation, i.e., (1) a new concrete culvert with bigger dimensions for a better run-off capacity, (2) a dry water pond for increased water capacity, and (3) a macadam basin with permeable asphalt. All three measures are expected to provide full protection regarding impacts on the traffic and the transport infrastructure at the site, and therefore reducing the expected socioeconomic cost to zero. The dry pond is a vegetative solution with either submerged grass or other ground surface, with little need for maintenance. In addition, one more reactionary measure is included, i.e., (4) increased pumping capacity combined with increased preparedness. This is, for example, achieved through investment in more pumps or improved distribution capacity of pumps to reduce the magnitude, and thereby the consequences, of a similar or more severe heavy precipitation event. In this study, those additional pumps are expected to reduce the time of the stop by half in case of flooding, therefore reducing the expected socioeconomic cost of the flood event to roughly half of the estimated costs without a measure. All of the measures will also contribute to reduce the probability of a landslide to occur. In the cost-benefit analysis below, we assume that the current return time of a potential landslide is 100 years, the socioeconomic cost of the landslide is 55 MSEK (based on the valuation in the previous section), and the preventive measures are set to reduce the risk to zero, while additional pumping does not reduce the risk at all.

To calculate the NPV of measures and find the most socioeconomic efficient preventive measure, parameters such as initial cost of construction, annual cost of maintenance, economic life span of measure, discount rate, and marginal cost of public funds (MCPF) are needed. Here, based on ASEK 7.0 [41], the discount rate was set to 3.5 percent and the MCPF to 1.3. In Table 2, the estimated costs for the four measures are summarised both for construction and annual maintenance during an economic life span of 40 years for each of the measures, the present value of costs, and finally the NPV. According to Table 2, the most socioeconomically efficient measure, under these conditions, is a new culvert, while improved pumping capacity is estimated to be least socioeconomically efficient. However, these results are not meant to establish the best solution for the local context, but rather to highlight the methodology, since both the effectiveness of the measures, the return times, and the cost parameters are based on rough estimations.

Using NPV-estimates that include the increased probability of the flooding event due to a changing climate over time (Equation (3)) results in increased NPV, in comparison to NPV-estimates based on a fixed 100-year return time (Table S5). Despite taking this change into account, the relative ranking among the preventive measures do not change since the relative benefits of the measures changed equally for all of the preventive measures. The improved pumping capacity contributed less to the relative benefit, not altering the relative ranking. The NPV of the preventive measures increases around five times by also taking into account the benefits related to reduced risks of landslides (Table 2). The probability of a flooding (due to similar or more intense precipitation event), and the probability of other events such as a landslide, is likely to increase over time, which may alter when in time an investment is beneficial. As seen in Table S6, applying Equation (4) and a return time change from 1000 to 100 years for flooding results in negative NPV:s for the four measures, when including only the flooding, i.e., the NPV is only positive for the three preventive measures when also including a landslide. Further, some of the measures where NPV are currently net negative might become net positive at a later investment time, as can be seen in Tables S7 and S8. However, if only considering flood events, given the parameters applied here, the investigated preventive measures are shown to either be net positive from the start or not at all (Table S7). Though, an investment not relevant for a flood event may become beneficial at a later occasion when also considering other risks such as a landslide. In addition to uncertainties in return time, several factors such as the discount factor may alter the results and are therefore included in the sensitivity assessment step.

#### Multi-Criteria Analysis

As not all costs are able to estimate in monetary terms, in addition to the cost-benefit analysis, a multi-criteria analysis (MCA) was performed for the three preventive measures and the one reactive ditto (Table S9). Additionally, more complex measures that are not included in the CBA above, such as bridges over low points, embankments, and land elevation changes, are collectively included to give a perspective of larger potential measures.

This MCA is based on Andersson-Sköld and Nyberg [33] and designed for the practices and needs of the Swedish Transport Administration. Based on the assessment conducted here, the ranking between the measures differs in the MCA compared to the monetary (NPV) estimated ranking. In the MCA, the dry water pond is the most beneficial alternative, twice as beneficial as the new culvert, the macadam solution, and the improved pumping capacity (Table S9). The more expensive solutions even provide a negative impact in total (Table S9). As the three preventive measures (culvert, dry water pond, and the macadam) scored rather similar in the monetary evaluation, the dry water pond solution seems to be the most relevant measure to undertake based on the MCA assessment. Here, the authors have conducted the assessment and set the weights of the different aspects considered in the assessment equal in relation to each other. In a real case assessment, it is important that several experts and relevant stakeholders should be involved in the assessment and the weighing of the importance of the different aspects considered. Such a broader expert and stakeholder involvement may contribute to alter the result of the combined monetary and non-monetary valuation, for example, aspects not considered in the MCA method applied may arise, such as responsibilities among different stakeholders. The results of incorporating the MCA in the assessment do, however, indicate the importance to have a holistic perspective in the assessment, both including monetary and non-monetary evaluations.

#### 3.2.4. Step 4. Sensitivity Analysis

The estimates in steps 1–3 are based on literature, available budget and procurement costs, expert experiences, and judgements as well as the authors own assumptions resulting in large uncertainties. However, we want to stress that the main reason for the case study is not to find the best preventive measure for that specific context, but rather to illustrate the method and exemplify how it can be applied. Here, the impacts of uncertainties are exemplified by assessing the estimated NPV of the flood related impact changes in investment and maintenance costs, changes in the measures lifetime, and changes in AADT, queueing time and discount rate.

The estimated damage cost related to the flooding is heavily influenced by AADT and the assumption of queueing time, ranging from 1.4 million SEK (75% AADT, 0 h of queueing) to 36.3 million SEK (125% AADT, 10 h of queueing) (Table S10). Moreover, the discount rate has a large impact on the estimated NPV, for example, changing it from 3.5 percent to zero results in increased NPV for all measures (e.g., from 3.5 to 7.8 MSEK for a new culvert). In addition, the estimate of when in time the measure is beneficial to conduct is changed (Tables S7 and S8), but not the internal ranking among the measures. It is worth remembering both present value of cost and benefits increase with zero discounting.

We also investigate the impact of other annual scaling factors, by applying a scaling factor to e.g., AADT or the evaluation of benefits related to aspects such as travel time, pollution, and traffic accidents, since the importance of the measures thereby may increase in the future for example due to growth in the economy and disposable income [41]. As those uncertainty factors interact with the expected present value of benefits, $PV_{B}$, we can incorporate them into a total change factor together with the discount rate of the present values of benefits:

(6) $$PV_{B}=\overset{k}{\underset{t=1}{\sum }}P*D*{\left(1+\alpha \right)}^{t}*{\left(1+\beta \right)}^{t}*{\left(1+r\right)}^{-t}=\overset{k}{\underset{t=1}{\sum }}P*D*\gamma^{t}$$

where:

α is the annual change in e.g., AADT,

β is the annual change in e.g., valuations, and

γ is the change factor:

$$\gamma =\frac{\left(1+\alpha \right)\left(1+\beta \right)}{1+r}$$

Both the change factor and the life span of the measure impacts the NPV (Table S11). The longer the economic life spans the higher the NPV. As seen in Table S12, the tested changes may either work by counteracting the discount rate, thereby almost cancelling each other out, or in the same direction and thereby alter the NPV. Again, however, not altering the internal ranking among the measures.

Another uncertainty is related to the costs of preventive measures. In Table 3, NPV-estimates of the four measures are shown when the present value of costs, $PV_{C}$, of both investment and maintenance are changed by ±25 percent relative to the applied costs in step 4 (Table 2). The resulting NPV-based ranking between the measures becomes less obvious due to some overlap in the resulting spans (Table 3).

In this case study, we have investigated the factors impacting the NPV of preventive measures. To assess the uncertainties and sensitivity of all aspects included, Monte Carlo simulations could be performed and is recommended for real case assessments. The goal analysis was here captured in the multicriteria analysis, but the aspects considered could be expanded to related to local, regional, and national goals and objectives. Finally, distribution analysis is assumed to have no net differences between measures.

## 4. Discussion

This article presents a framework that shall be applied to support how to prioritize among climate risk reduction measures for the road and railroad systems. It includes the full effect-chain, from the identification of hazards to risk analysis and identification and valuation of potential measures. It also includes which methods can be applied for assessing the probability, consequence, and effect of potential risk reduction measures, as well as when measures are cost-effective to undertake. The framework thereby contributes to a more systematic and effective risk management process. Such a systematic and supporting process may also contribute to increase the common knowledge, and promote co-operation among various stakeholders, which currently needs to be improved, as stated by Leiren et al. [22]. To develop and apply the framework, several methods needed to be applied. That includes previous frameworks and methods already presented in the literature, expert experiences, and their expressed needs regarding climate risks today and in a changing climate. As climate risks, and adaption of the infrastructure, is complex, there is a need of several methods and data (summarised in Table 1) to be applied through different steps as in the framework. Here, we illustrate this by applying the framework on a case study.

The aim of the framework is for it to be used as a basis for identifying the need for introducing risk reduction measures. If so, the framework shall also be further applied for prioritization of potential risk reduction measures, and where and when to undertake it. The framework is generic and scientifically based. However, some values and methods that are suggested (Table 1) are based on expert knowledge and experiences from the Swedish context, and the Swedish rail and road system. Therefore, instead of applying Swedish internalization values, ASEK 7.0 [41], country/site specific values and methods should be used in the framework. Further, even if the framework consists of steps that are consecutive, the process of applying the framework should be iterative.

Based on minor interviews with the STA, they regard it applicable, systematic, and useful to assess the complicated climate risks and potential effects of risk reduction measures. The generality of the framework is also applicable for evaluating climate change adaptation measures in other sectors. However, this may require contextual adaption. When applied in the case study, the method worked well, but we also identified some important knowledge gaps that needs to be closed to be able to make more robust assessments and analyses. Further, there is a need of development towards better and more covering data as well as a better understanding of the full effect-relationship-chain, as also stated by Stamos et al. [3]. This is especially important regarding availability of statistical information on return times and down-scaled projections. There is also a need to make annual probability estimates in a changing climate. For the latter, there is a need to be able to make better estimates of the landslide probability due to projected changes in precipitation, hydrological patterns, and other changes in the soil such as the soil temperature and soil biology in soft and sensitive soils [27]. Further, the NPV-assessment strongly depends on whether consequential effects are included or not. The occurrence of landslides, as a subsequent effect, depends on the type of weather-related event causing the delay, as well as other factors.

A data-driven approach to assess the impacts of extreme weather events on passenger flows was recently developed and applied on passenger flow-shifts in a European small-scale network [3]. Such a method is highly relevant to apply in step 2 of the framework presented in this paper. Such a data driven approach would require data-compilations of passenger flows on local, county, and national level, which would require access to such databases. The investment and maintenance costs, as well as other costs and benefits of a measure, shall be estimated for current climate as well as projected climatic changes. Mostly, the estimated probability of a medium- and long-term perspective is achieved by adding the expected change to the current return times. This is a rather simple way, which could be further developed, for example by local climate projection scenarios. Such scenarios are applied by, for example, the Gothenburg city, Sweden, for planning based on projected flood risk [81]. The framework described here includes how to identify cost-effective measures taking projected climate change into account. The framework would, however, benefit from evolving into a GIS-based system, based on available local and county level statistical weather data, and climate change projections. A GIS-based system would also allow coupling to existing databases and systems, such as soil and land-use information and topography.

Even though there are methods available for assessing costs related to the passenger flow changes, there is also a need to better predict the costs of climate related risks. An example could be to include impacts that alter the access to socially important activities due to delays and cancelled traffic, or environmental impacts with and without risk mitigation measures. This was also included in the framework presented here, otherwise often omitted. To assess the costs and benefits of potential measures, the effectivity of the measure needs to be known. Further, the environmental, social, and socioeconomic impacts need to be evaluated to achieve the most sustainable solutions. Here, we applied both monetary (NPV) and non-monetary (MCA) assessments for such an evaluation, where the case study illuminates the impact of including such a more holistic perspective rather than a pure monetary evaluation.

The analysis in the final step of the framework shall ensure that the measures contribute to a sustainable development. Here, we therefore suggest that the analysis includes a goal-analysis related to the SDG, translated to the actual context. This assessment can be included in an integrated CBA and MCA, preferably involving relevant stakeholders, planners, and decision makers. The weighting procedure also benefits from including the same representatives. The results from those processes depend on who participates and who carries out the analysis. A flexible project management [21], including collaboration and explorative learning, will improve the results and smoothen the planning and decision process in which the outcomes from the framework application will be used. Flexible project management is important in any complex projects (technically or organizationally), as stated by Eriksson et al. [21]. Though, as forwarded by Daly [71], the environmental aspects must be considered, as the frame must be set from a sustainable development perspective. As the social impacts depend on who is affected, this framework also assesses how the potential risk reduction measures impact various groups of members in the society through a distribution analysis. For a holistic and sustainable adaptation, the need of impacts and solutions on different spatial scales also need to be incorporated [82], as considered in the MCA applied here. The results of the MCA and the weighing procedure depend on who is involved and uncertainties in available data and methods. Sensitivity analysis is therefore crucial. Here, we included variations in AADT, discount rate and a more unclear uncertainty factor. Especially the absolute NPV was altered, also the time for when an investment was found beneficial varied, while the internal ranking of measures was not. For real cases, we recommend even more thorough analysis, for example using Monte Carlo simulations.

In summary, the framework presented here includes all the steps in the cause-effect-relationship chain from hazard identification to prioritization including not only a sensitivity analysis but also a goal and distribution analysis, and when in time a measure is relevant to undertake. The framework is systematic and generic, but the assessments and valuations need to be conducted, taking site specific considerations into account.

## 5. Conclusions

This article has presented a framework that shall be applied to prioritize among climate risk reduction measures for the road and railroad systems. It includes the full effect-chain, from the identification of hazards to risk analysis and identification, valuation of potential measures, and when in time to undertake a measure. It includes information on potential risk reduction measures related to impacts that may be caused by weather related events expected to increase due to climate change (temperature, sea level rise, changes in precipitation). It considers potential consequences such as road surface impacts, solar curves on rail, flooding, erosion, and the related social and socioeconomic costs. The framework also includes how to assess the probability and the consequences of specific weather-related events in a changing climate, how to assess the effect of risk reduction measures, both in monetary and non-monetary terms, and a final step with a sensitivity analysis as well as a goal and distribution analysis.

The framework is relevant, generic, and applicable for climate adaptation of road and rail infrastructure. Furthermore, it could easily be adapted and applied in other sectors. Regardless of the sector, the assessments and valuations need to be conducted, accounting for considerations. The framework can be used based on currently available data and methods for assessing risks and effects of risk reduction measures, as well as being applied and further adapted as more data and methods are being developed. It is systematic and useful to assess the complicated risks and effects of risk reduction measures which need to be taken in a changing climate.

## Figures and Tables

**Table 1:** Climate-change related weather events and potential consequences considered, potential risk reduction measures and methods, data and investigations identified to be applied for the risk and effect assessments.

Climate-Related Events	Example of Data to Be Used	Potential Consequences	Subsequent Potential Consequences (Figure 1)	Methods and Data Required for Ex-Ante Assessment of Potential Consequences ^3^	Potential Risk Reduction Measures	Improvements Needed for More Robust Effect Estimates
Temperature and radiation related events	Statistical data and climate change projections ^1^ on: Mean temperatures (air and radiation) (including locations with shade/high radiance) Extreme temperatures Temperature zero-crossings	Bitumen bound layers e.g.: Ruts Bleeding asphalt	Reduced speeds Increase in traffic accidents (injures/fatalities, vehicle damages, queues, and reduced speed, increased costs due increased maintenance)	Experience and statistics on previous events and performed maintenance, reported needs, measures and forecasts as described in guiding material (e.g., [46]) complemented with inventories, solar radiant temperature simulations with e.g., Solveig [47] and classification of maintenance needs for different types of roads. The latter is used for prioritization of maintenance.	Maintenance: Add aggregate of different grain size on bleeding asphalt will reduce slipperiness Cool the asphalt with water to prevent bleeding (not to be used once bleeding has occurred) [8] Substitute bleeding asphalt with new [8]	Development and criteria for classifying and prioritizing maintenance needs taking climate change into account
		Solar curves on rail		Method and criteria for classifying and prioritizing maintenance needs. Inventory and classification of current maintenance needs with respect to buckling. The inventory is based on existing inventories, new general and site-specific inventories. Reporting complemented by ex-ante solar radiant temperature simulations with e.g., Solveig [47].	To minimize solar cures, continuous maintenance is required including verifying that there is sufficient and well packed ballast, maintaining and (when necessary) replacing rail fortifications and sleepers.	
		Temperature-related impacts on functionalities of switches, bridges, and tunnels		Compilation of information on previous events, inventories of current functionality	Continuous inventory and maintenance. E.g., install a digital monitoring system that: Warns when joints become too dense or replace materials to more heat resistant ones.	Increased inspections, both in frequency during high temperature events as well as in content, e.g., to include expansion of bride joints. Procedures and methods for this need to be developed and included in contracts and/or development of automatic monitoring systems. Examples of corrosion warning systems can be found in McCarter and Vennesland [48] and a variety of monitoring sensors and systems tested for bridges are provided in [49].
Fire risks (increase in temperature causes evaporation, and the drier the environment the faster the fire spreads)	Projections on local/county level fire risk [50]	Initiation of a fire due to sparking caused by road accidents, working machines and vehicle fleets used by road contractors or parts of the facility	Potential road damage requiring restoration, such as planing of the road surface, restoration of the bearing capacity of the sub-base and base layers of the road, trenching and drum replacements Stop in traffic Redirecting traffic (which will cause reduced speeds and increased risks for accidents) Reduced fire-fighting capacity	Reduce the risk of sparking, spreading, and improve the ability to extinguish and control a fire: Ensure a fireproof machine and vehicle fleet among contractors Regular review and maintenance of vehicles (road and rail) and machinery Limit admissibility when road and rail work may be carried out Ensure that the minimum necessary access and functionality of airports and roads all over the country Ensure the presence of a sufficiently good redirecting capacity in connection with fire. Ensure good preparedness and interaction with other authorities, organizations, and actors Measures that may need to be taken to restore the road are the planing of road surface, restoration of the bearing capacity of the sub-base and base layers of the road, trenching and drum replacements		There is a need to better understand and identify measures to reduce emergence and risk of fire spreading and how to increase efficiency in the event of a fire. Such a project should involve several actors (the Swedish Transport Agency, Civil contingencies authorities, the Swedish Meteorological and Hydrological Institute (SMHI) and the STA), other stakeholders and researchers.
		Other causes of fire than the transport infrastructure				
Strong winds	Statistical data and climate change projections ^1^ on: Mean wind Maximum wind gusts	Damages on constructions or damages caused by blown off parts of constructions Vehicles blown off road Blown off debris from vehicles or road/nearby road equipment Preventive closings of bridges, rail, and road	Accidents Stop in traffic Reduced speeds Redirection/change of transport mode	To estimate the expected impact of strong winds and the measures that are relevant, taking the expected change in climate into account, requires awareness of the necessity for action today. An inventory of the requirement to strengthen or protect the material used in the bridge construction to withstand future stresses from strong winds, increased moisture and violent storm surges therefore need to be made for the current situation, to get an idea of the need for action, the cost of action and the expected impact, taking the current situation into account.		
Snowstorm	Statistical data and climate change projections ^1^ on: Temperature Precipitation as snow Mean wind Maximum wind gusts	Reduced visibility Drifting snow Increased sliding risk, Preventive closings of bridges, rail, and road	Accidents Stop in traffic Reduced speeds Redirection/change of transport mode	To estimate the expected impacts and the measures that are relevant, taking the expected change in climate into account, requires awareness of the necessity for action today and improved knowledge on expected projections.		
Changed ground conditions in temperature and humidity	Statistical data and climate change projections ^1^ relevant to estimate ground temperature and humidity patterns including number of temperature zero-crossings. Current and expected changes in water table and groundwater formation. [51]	Buoyancy Subsidence and horizontal deformation	Reduced speeds Preventive redirection/change of transport mode	Information regarding the status of roads and rails as well as the functionality of drums and current flow capacity in trenches, culverts, drums and pipes.	Improve maintenance and bearing capacity, combined with inventories of asphalt damage to identify weaknesses.	Current variations in groundwater levels need to be studied in more detail to be able to estimate the potential impacts of projected changes. This is needed to understand and assess potential effects of the changes. Further research is needed to be able to quantify and describe the processes and properties that will be affected.
Sea level rise	Statistical data, including air pressure, wind velocity and wind direction, and climate change projections ^1^ on sea water levels	Flooding Erosion Landslides	Accidents Stop in traffic Reduced speeds Redirection/change of transport mode	*Flooding* Models and modules as included in MikeShe^®^ Using (GIS) information: Topographical information, incl. high road banks and bluespots Detailed soil maps Proportion of hard surface Vegetation incl. green coverage and crown coverage (e.g., [24]), root depth and plant coefficient [52] taking seasonality into account) Surface rawness coefficient [53,54] in [52] Information on ditches, streams and their flows and capacity (may require inventorying) Knowledge and capacity of culverts (location, dimensions), drums and wires (inventory may be required) *Erosion and landslides* When available, field data for single sections can be used to assess current bottom and bank erosion and to validate, and assess the plausibility of, values and methods used for ex-ante calculations [55]. Data needed for estimates of erosion, currently and projected: Hydrological statistics: surface water levels, annual average and medium water flow, water flow with 100-year return time and maximum flow. Expected hydrological changes based on simulations (altered precipitation, temperature and run-off e.g., MikeShe, HBV et al. models). Soil/sediment information (potential share of silt and clay fraction). Geotechnical field measurements. Calculation of bottom erosion e.g., as described in [55] For calculations of bank erosion, models such as BSTEM ^2^ can be used. This requires geometry, flow characteristic, soil layer thickness and soil layer characteristics. Erosion affects the landslide probability and depend on several additional factors (e.g., slope height and inclination, soil layers strength characteristics, groundwater level, pore water pressure, and the load [18].	*Flooding* Increase preparedness and management to manage and maintain traffic and electrical installations during flooding and temporary measures such as: Planned and prepared redirecting of traffic or alternative transport modes. Preparedness for use of temporary bridge, additional pumps, temporary protection Increase dewatering capacity Install stationary pumps. This requires accessible and functioning external reservoirs, wells and ditches. Increased maintenance of drums, wells and pipes at low points (this requires inventories). Water retardation and flood-allowing surfaces through Increased vegetation, stormwater ponds, overland flow surfaces Stationary solutions, such as increased lowest height, protection walls and dikes *Erosion and landslides* Increased maintenance of existing erosion protection Installation of hard erosion protection (gravel and stone, concrete blocks, gabions and steel or wood piles) Installation of soft erosion protection (grass, grass and shrubs, trees and shrubs, coconut mats or geotextile with vegetation, dead plant material) Combined erosion prevention (revetments with vegetation, gabions and vegetation, concrete blocks with vegetation, wooden piles with vegetation, logs, log walls, or dead wood, vegetation and stones to reduce water speed and control ice and different types of flow change and assault erosion protection Flattening of the geometry of the slope. De-excavation of the ground in the upper part of the slope Counter fill lower part of the slope Lowering or restricting pore pressure Lime/cement pillars	*Erosion and landslides* Bathymetric measurements are used for reliable assessments of ongoing erosion and to validate methods and parameters and values used in calculations. Unfortunately, such measurement data is usually lacking, which is why only calculations must be used To assess the landslide probability per unit of time (e.g., per year) there are promising methods available but they need to be developed and validated (lab. field) [56]. To estimate the landslide probability in a changing climate, the processes need to be better understood: How does the change in average temperature affect the physical and chemical properties at different depths and what does this mean for the hydrological, geological and geotechnical characteristics? How quick are changes expected to occur and how will this impact erosion and landslide probability? There is a lack on how to describe the soil processes in unsaturated zones and how the properties will change in a changing climate. E.g., how will changing drought precipitation cycles affect dry crust and cracking? How will changes in frost/thaw cycles and other climatic changes impact rock cracking?
Increase in precipitation and higher amplitude in water table variations	Statistical data and climate change projections ^1^ on: Mean precipitation Extreme precipitation Snow depth Current and expected changes in water table and groundwater formation [51]					

^1^ Statistical data for Swedish local/county and national level can be achieved from https://www.smhi.se/klimatet-da-och nu/klimatindikatorer/ (accessed on 28 September 2021) and climate change projections on local/county level can be obtained from https://www.smhi.se/klimat/framtidens-klimat/klimatscenarier (accessed on 28 September 2021) unless other data source provided. ^2^ Local information on previous sea water levels under events with temporary increased sea water level (air pressure and wind speed and direction). ^3^ The magnitude of the impact as well as its cost also depends on the traffic flow (number of vehicles, type of vehicle) and the time for a closure or redirection and repair costs as illustrated by the equations in Supplementary Materials.

**Table 2:** Cost benefit inputs, present value of benefits and costs, and net present value of four measures, with increasing probability of flooding over time (100-year event to 10-year event), SEK in 2017-year prices.

	New Culvert	Dry Water Pond	Macadam Basin w/Permeable Asphalt	Additional Pump Capacity
Investigated economic timespan	40 years	40 years	40 years	40 years
Lifetime of intervention	≥40 years	≥40 years	≥40 years	<10 years (but investment estimated as annual cost corresponding to 20,000 per year
Investment cost (excl. MCPF)	200,000	500,000	1,000,000	
Maintenance cost (excl. MCPF)	10,000	5000	0	
Expected damage reduction	100%	100%	100%	50%
Present value benefits (flooding)	4,003,000	4,003,000	4,003,000	2,001,000
Present value benefits (flooding and landslide)	15,748,000	15,748,000	15,748,000	2,001,000
Present value costs	538,000	789,000	1,300,000	555,000
Net present value (flooding)	3,465,000	3,214,000	2,703,000	1,446,000
Net present value (flooding and landslide)	15,211,000	14,959,000	14,448,000	1,446,000

**Table 3:** Present value of benefits, costs, and net present value when applying ±25% investment and maintenance costs, in relation to costs in Table 2, SEK in 2017-year prices.

	New Culvert	Dry Water Pond	Macadam Basin w/Permeable Asphalt	Additional Pump Capacity
Present value benefits (flooding)	4,003,000	4,003,000	4,003,000	2,001,000
Present value costs	403,000–672,000	592,000–986,000	975,000–1,625,000	416,000–694,000
Net present value (flooding)	3,331,000–3,600,000	3,017,000–3,411,000	2,378,000–3,028,000	1,307,000–1,585,000

## Data Availability

Data is contained within the article or Supplementary Materials.

## Supplementary Materials

The following are available online at https://www.mdpi.com/article/10.3390/ijerph182312314/s1: Table S1. Experts interviewed, Table S2. Traffic input regarding annual average daily traffic (AADT), traffic affected by flooding, additional travel distance and time, Table S3. Shadow prices for marginal costs of traffic in rural areas for passenger car (PC), passenger car in commercial traffic (PCC), lorry without trailer (L) and lorry with trailer (LT). The unit is either SEK per vehicle kilometer or SEK per vehicle hour, 2017-year prices [41], Table S4. Summary of estimated socioeconomic costs and relative share, per item due to the case study heavy precipitation event, Table S5. Present value of benefits and costs and net present value of four measures, without increasing probability of flooding over time (100-year event), SEK in 2017-year prices, Table S6. Present value of benefits and costs and net present value of four measures, with increasing probability of flooding over time (1000-year event to 100-year event), SEK in 2017-year prices, Table S7. First year of positive net present value over 40-year period, and respective net present value, based on damage cost of flooding and investment and maintenance costs, discount rate at 3.5%, year and SEK, Table S8. First year of positive net present value over 40-year period, and respective net present value, based on damage cost of flooding and investment and maintenance costs, discount rate at 0%, year and SEK, Table S9. Multi-criteria analysis of preventive and reactionary measures. The impact values are given on a scale from -5 (very unwanted impact) to +5 (very wanted impact) and in relation to not undertake any measure, Table S10. Socioeconomic cost of flooding based on different values of AADT and queueing, SEK in 2017-year prices, Table S11. Net present value of a 1,000,000 SEK damage reduction due to preventive measure, based on economic life span and change factor, Table S12. Change factor based on valuation change factor and discount rate, Figure S1. Example of risk matrix for natural disasters (flooding, landslide, and erosion) within STA.
