# Supplementary material for: A Framework for Identification, Assessment and Prioritization of Climate Change Adaptation Measures for Roads and Railways
Source: Int J Environ Res Public Health. 2021 Nov 23;18(23):12314. doi: 10.3390/ijerph182312314 (PMC8657026; doi:10.3390/ijerph182312314)
Supplement: Supplementary file 1 [file ijerph-18-12314-s001.zip › ijerph-1419370-Supplementary.pdf]

International Journal of
Environmental Research and Public Health

MDPI

Supplementary material

Supporting information

The supporting material includes:

- Table S1. Experts interviewed
- Table S2. Traffic input regarding annual average daily traffic (AADT), traffic affected by flooding, additional travel distance and time.
- Table S3. Shadow prices for marginal costs of traffic in rural areas for passenger car (PC), passenger car in commercial traffic (PCC), lorry without trailer (L) and lorry with trailer (LT). The unit is either SEK per vehicle kilometer or SEK per vehicle hour, 2017-year prices [41].
- Table S4. Summary of estimated socioeconomic costs and relative share, per item due to the case study heavy precipitation event.
- Table S5. Present value of benefits and costs and net present value of four measures, without increasing probability of flooding over time (100-year event), SEK in 2017-year prices.
- Table S6. Present value of benefits and costs and net present value of four measures, with increasing probability of flooding over time (1000-year event to 100-year event), SEK in 2017-year prices.
- Table S7. First year of positive net present value over 40-year period, and respective net present value, based on damage cost of flooding and investment and maintenance costs, discount rate at 3.5%, year and SEK.
- Table S8. First year of positive net present value over 40-year period, and respective net present value, based on damage cost of flooding and investment and maintenance costs, discount rate at 0%, year and SEK.
- Table S9. Multi-criteria analysis of preventive and reactionary measures. The impact values are given on a scale from -5 (very unwanted impact) to +5 (very wanted impact) and in relation to not undertake any measure.
- Table S10. Socioeconomic cost of flooding based on different values of AADT and queueing, SEK in 2017-year prices.
- Table S11. Net present value of a 1,000,000 SEK damage reduction due to preventive measure, based on economic life span and change factor.
- Table S12. Change factor based on valuation change factor and discount rate.

- Figure S1. Example of risk matrix for natural disasters (flooding, landslide, and erosion) within STA.

Table S1. Experts interviewed.

Role interviewee	Organization
Road Specialist, Road Coordinator and Representative of Operations and maintenance. Responsible for the texts and assessments of expected climate change impacts on roads in the National Climate and Vulnerability Investigation (SOU, 2007:60a).	Swedish Transport Administration (STA)
Specialist support on geotechnical engineering in the STA. Member of the National Delegation on Landslides.	STA, Swedish National Delegation on Landslides
Regional Urban Planner. Developed a GIS-based climate and vulnerability analysis tool for the STA.	STA
Specialist support on dewatering in the STA.	STA
Rail operations manager	STA
Rail operation leader	STA
Train conductor	STA
Road Traffic Management Stockholm	STA
Geotechnical engineer, regulations, maintenance, and development issues	STA
Responsible for STA climate adaptation strategy	STA
National economists at STA (2)	STA
Senior Researcher, wheel- and rail impacts	The Swedish National Road and Transport Research Institute (VTI) and Chalmers University of Technology
Senior researcher and engineer on operation and maintenance of railways, also teaches the railway engineers at the,	VTI and Transport Administration School
Professor of Geotechnical Engineering	Chalmers University of Technology

Table S2. Traffic input regarding annual average daily traffic (AADT), traffic affected by flooding, additional travel distance and time.

	**Passenger cars/ Light traffic**	**Freight travel/ Heavy traffic**
Annual average daily traffic (AADT) (vehicles)	12,400	1,600
Traffic affected, queuing 5 hours (vehicles)	4,926	510
Traffic affected, rerouting 11 hours (vehicles)	7,299	782
Additional travel distance per vehicle, rerouting (km)	12.8	14.8
**Additional travel distance, total (vehicle km)**	**158,720**	**23,680**
Additional travel time per vehicle, rerouting (h)	0.25	0.25
Additional travel time, queuing (vehicle h)	12,315	1,274
Additional travel time, rerouting (vehicle h)	1,825	196
**Additional travel time, total (vehicle h)**	**14,139**	**1,470**

Table S3. Shadow prices for marginal costs of traffic in rural areas for passenger car (PC), passenger car in commercial traffic (PCC), lorry without trailer (L) and lorry with trailer (LT). The unit is either SEK per vehicle kilometer or SEK per vehicle hour, 2017-year prices [41].

Socioeconomic cost	Unit	PC	PCC	L	LT
Freight value of time costs (excl. VAT)	SEK per vehicle hour	-	7.63	19.08	88.97
Maintenance and repairs: Payroll cost (PC/PCC); Service and reparations (L/LT)		0.14	0.33	1.16	1.19
Value of time travel costs		637	1,519	1,168	973
Additional road maintenance and reinvestment costs	SEK per vehicle kilometer	0.031	0.031	0.434	0.434
Capital cost: Depreciation		0.71	2.23	4.90	2.25
Fuel costs (excl. VAT and tax)		1.02	0.70	1.86	2.62
Maintenance and repairs: Component wear and tear (PC/PCC); Tires (L/LT)		0.16	0.51	0.48	0.94
Marginal cost: Additional pollution (CO2)		0.94	0.94	3.05	4.47
Marginal cost: Additional pollution (other pollutants)		0.001	0.001	0.005	0.005
Marginal cost: Traffic accidents		0.024	0.024	0.815	0.815

Table S4. Summary of estimated socioeconomic costs and relative share, per item due to the case study heavy precipitation event.

	Socioeconomic cost (SEK in 2017-year prices)	Share of total cost
Value of time travel costs (queuing)	9,315,129	83.1%
Value of time travel costs (rerouting)	1,382,309	12.3%
Marginal cost: Additional pollution (CO2)	132,378	1.2%
Capital cost: Depreciation	119,739	1.1%
Fuel costs (excl. VAT and tax)	118,787	1.1%
Freight value of time costs (excl. VAT)	76,928	0.7%
Maintenance and repairs: Component wear and tear / Tires	26,417	0.2%
Maintenance and repairs: Payroll cost / Service and reparations	13,800	0.1%
Marginal cost: Traffic accidents	11,680	0.1%
Additional road maintenance and reinvestment costs	7,922	0.1%
Marginal cost: Additional pollution (other pollutants)	151	0.0%
**Total**	**11,205,239**	**100%**

Table S5. Present value of benefits and costs and net present value of four measures, without increasing probability of flooding over time (100-year event), SEK in 2017-year prices.

	New culvert	Dry water pond	Macadam basin w/ permeable asphalt	Additional pump capacity
**Present value benefits (flooding)**	2,392,887	2,392,887	2,392,887	1,196,443
**Present value benefits (flooding and landslide)**	14,138,177	14,138,177	14,138,177	1,196,443
**Present value costs**	537,616	788,808	1,300,000	555,232
**Net present value (flooding)**	**1,855,271**	**1,604,079**	**1,092,887**	**641,212**
**Net present value (flooding and landslide)**	**13,600,561**	**13,349,369**	**12,838,177**	**641,212**

Table S6. Present value of benefits and costs and net present value of four measures, with increasing probability of flooding over time (1000-year event to 100-year event), SEK in 2017-year prices.

	New culvert	Dry water pond	Macadam basin w/ permeable asphalt	Additional pump capacity
Present value benefits (flooding)	400,283	400,283	400,283	200,142
Present value benefits (flooding and landslide)	12,145,573	12,145,573	12,145,573	200,142
Present value costs	537,616	788,808	1,300,000	555,232
**Net present value (flooding)**	**-137,333**	**-388,525**	**-899,717**	**-355,090**
**Net present value (flooding and landslide)**	**11,607,957**	**11,356,765**	**10,845,573**	**-355,090**

Table S7. First year of positive net present value over 40-year period, and respective net present value, based on damage cost of flooding and investment and maintenance costs, discount rate at 3.5%, year and SEK.

Investment cost (excl. MCPF)	Maintenance cost (excl. MCPF)	Damage cost (SEK in 2017-year prices)					
		1,000,000	25,000,000	50,000,000	100,000,000	500,000,000	1,000,000,000
**10,000**	**10,000**	0 (66,613)	0 (8,640,095)	0 (17,570,807)	0 (35,432,229)	0 (178,323,609)	0 (356,937,833)
**10,000**	**100,000**	- (-)	0 (6,141,552)	0 (15,072,263)	0 (32,933,686)	0 (175,825,065)	0 (354,439,290)
**10,000**	**1,000,000**	- (-)	- (-)	- (-)	0 (7,948,251)	0 (150,839,631)	0 (329,453,855)
**10,000**	**10,000,000**	- (-)	- (-)	- (-)	- (-)	- (-)	0 (79,599,509)
**100,000**	**10,000**	- (-)	0 (8,523,095)	0 (17,453,807)	0 (35,315,229)	0 (178,206,609)	0 (356,820,833)
**100,000**	**100,000**	- (-)	0 (6,024,552)	0 (14,955,263)	0 (32,816,686)	0 (175,708,065)	0 (354,322,290)
**100,000**	**1,000,000**	- (-)	- (-)	- (-)	0 (7,831,251)	0 (150,722,631)	0 (329,336,855)
**100,000**	**10,000,000**	- (-)	- (-)	- (-)	- (-)	- (-)	0 (79,482,509)
**500,000**	**10,000**	- (-)	0 (8,003,095)	0 (16,933,807)	0 (34,795,229)	0 (177,686,609)	0 (356,300,833)
**500,000**	**100,000**	- (-)	0 (5,504,552)	0 (14,435,263)	0 (32,296,686)	0 (175,188,065)	0 (353,802,290)
**500,000**	**1,000,000**	- (-)	- (-)	- (-)	0 (7,311,251)	0 (150,202,631)	0 (328,816,855)
**500,000**	**10,000,000**	- (-)	- (-)	- (-)	- (-)	- (-)	0 (78,962,509)
**1,000 000**	**10,000**	- (-)	0 (7,353,095)	0 (16,283,807)	0 (34,145,229)	0 (177,036,609)	0 (355,650,833)
**1,000 000**	**100,000**	- (-)	0 (4,854,552)	0 (13,785,263)	0 (31,646,686)	0 (174,538,065)	0 (353,152,290)
**1,000 000**	**1,000,000**	- (-)	- (-)	- (-)	0 (6,661,251)	0 (149,552,631)	0 (328,166,855)
**1,000 000**	**10,000,000**	- (-)	- (-)	- (-)	- (-)	- (-)	0 (78,312,509)
**10,000 000**	**10,000**	- (-)	- (-)	0 (4,583,807)	0 (22,445,229)	0 (165,336,609)	0 (343,950,833)
**10,000 000**	**100,000**	- (-)	- (-)	0 (2,085,263)	0 (19,946,686)	0 (162,838,065)	0 (341,452,290)
**10,000 000**	**1,000,000**	- (-)	- (-)	- (-)	- (-)	0 (137,852,631)	0 (316,466,855)
**10,000 000**	**10,000,000**	- (-)	- (-)	- (-)	- (-)	- (-)	0 (66,612,509)
**100,000 000**	**10,000**	- (-)	- (-)	- (-)	- (-)	0 (48,336,609)	0 (226,950,833)
**100,000 000**	**100,000**	- (-)	- (-)	- (-)	- (-)	0 (45,838,065)	0 (224,452,290)
**100,000 000**	**1,000,000**	- (-)	- (-)	- (-)	- (-)	0 (20,852,631)	0 (199,466,855)
**100,000 000**	**10,000,000**	- (-)	- (-)	- (-)	- (-)	- (-)	- (-)
**250,000 000**	**10,000**	- (-)	- (-)	- (-)	- (-)	- (-)	0 (31,950,833)
**250,000 000**	**100,000**	- (-)	- (-)	- (-)	- (-)	- (-)	0 (29,452,290)
**250,000 000**	**1,000,000**	- (-)	- (-)	- (-)	- (-)	- (-)	0 (4,466,855)
**250,000 000**	**10,000,000**	- (-)	- (-)	- (-)	- (-)	- (-)	- (-)
**300,000 000**	**10,000**	- (-)	- (-)	- (-)	- (-)	- (-)	- (-)
**300,000 000**	**100,000**	- (-)	- (-)	- (-)	- (-)	- (-)	- (-)
**300,000 000**	**1,000,000**	- (-)	- (-)	- (-)	- (-)	- (-)	- (-)
**300,000 000**	**10,000,000**	- (-)	- (-)	- (-)	- (-)	- (-)	- (-)

Table S8. First year of positive net present value over 40-year period, and respective net present value, based on damage cost of flooding and investment and maintenance costs, discount rate at 0%, year and SEK.

Investment cost (excl. MCPF)	Maintenance cost (excl. MCPF)	Damage cost (SEK in 2017-year prices)					
		1,000,000	25,000,000	50,000,000	100,000,000	500,000,000	1,000,000,000
**10,000**	**10,000**	0 (229,115)	0 (18,519,886)	0 (37,572,773)	0 (75,678,546)	0 (380,524,728)	0 (761,582,455)
**10,000**	**100,000**	- (-)	0 (13,839,886)	0 (32,892,773)	0 (70,998,546)	0 (375,844,728)	0 (756,902,455)
**10,000**	**1,000,000**	- (-)	- (-)	19 (1,000,683)	0 (24,198,546)	0 (329,044,728)	0 (710,102,455)
**10,000**	**10,000,000**	- (-)	- (-)	- (-)	- (-)	18 (82,758)	0 (242,102,455)
**100,000**	**10,000**	0 (112,115)	0 (18,402,886)	0 (37,455,773)	0 (75,561,546)	0 (380,407,728)	0 (761,465,455)
**100,000**	**100,000**	- (-)	0 (13,722,886)	0 (32,775,773)	0 (70,881,546)	0 (375,727,728)	0 (756,785,455)
**100,000**	**1,000,000**	- (-)	- (-)	19 (883,683)	0 (24,081,546)	0 (328,927,728)	0 (709,985,455)
**100,000**	**10,000,000**	- (-)	- (-)	- (-)	- (-)	19 (10,006,826)	0 (241,985,455)
**500,000**	**10,000**	25 (23,698)	0 (17,882,886)	0 (36,935,773)	0 (75,041,546)	0 (379,887,728)	0 (760,945,455)
**500,000**	**100,000**	- (-)	0 (13,202,886)	0 (32,255,773)	0 (70,361,546)	0 (375,207,728)	0 (756,265,455)
**500,000**	**1,000,000**	- (-)	- (-)	19 (363,683)	0 (23,561,546)	0 (328,407,728)	0 (709,465,455)
**500,000**	**10,000,000**	- (-)	- (-)	- (-)	- (-)	19 (9,486,826)	0 (241,465,455)
**1,000,000**	**10,000**	- (-)	0 (17,232,886)	0 (36,285,773)	0 (74,391,546)	0 (379,237,728)	0 (760,295,455)
**1,000,000**	**100,000**	- (-)	0 (12,552,886)	0 (31,605,773)	0 (69,711,546)	0 (374,557,728)	0 (755,615,455)
**1,000,000**	**1,000,000**	- (-)	- (-)	20 (747,110)	0 (22,911,546)	0 (327,757,728)	0 (708,815,455)
**1,000,000**	**10,000,000**	- (-)	- (-)	- (-)	- (-)	19 (8,836,826)	0 (240,815,455)
**10,000,000**	**10,000**	- (-)	0 (5,532,886)	0 (24,585,773)	0 (62,691,546)	0 (367,537,728)	0 (748,595,455)
**10,000,000**	**100,000**	- (-)	0 (852,886)	0 (19,905,773)	0 (58,011,546)	0 (362,857,728)	0 (743,915,455)
**10,000,000**	**1,000,000**	- (-)	- (-)	30 (1,195,338)	0 (11,211,546)	0 (316,057,728)	0 (697,115,455)
**10,000,000**	**10,000,000**	- (-)	- (-)	- (-)	- (-)	20 (7,471,098)	0 (229,115,455)
**100,000,000**	**10,000**	- (-)	- (-)	- (-)	30 (1,870,676)	0 (250,537,728)	0 (631,595,455)
**100,000,000**	**100,000**	- (-)	- (-)	- (-)	31 (27,353)	0 (245,857,728)	0 (626,915,455)
**100,000,000**	**1,000,000**	- (-)	- (-)	- (-)	- (-)	0 (199,057,728)	0 (580,115,455)
**100,000,000**	**10,000,000**	- (-)	- (-)	- (-)	- (-)	30 (11,953,382)	0 (112,115,455)
**250,000,000**	**10,000**	- (-)	- (-)	- (-)	- (-)	0 (55,537,728)	0 (436,595,455)
**250,000,000**	**100,000**	- (-)	- (-)	- (-)	- (-)	0 (50,857,728)	0 (431,915,455)
**250,000,000**	**1,000,000**	- (-)	- (-)	- (-)	- (-)	0 (4,057,728)	0 (385,115,455)
**250,000,000**	**10,000,000**	- (-)	- (-)	- (-)	- (-)	- (-)	7 (8,553,565)
**300,000,000**	**10,000**	- (-)	- (-)	- (-)	- (-)	2 (2,674,546)	0 (371,595,455)
**300,000,000**	**100,000**	- (-)	- (-)	- (-)	- (-)	3 (4,330,032)	0 (366,915,455)
**300,000,000**	**1,000,000**	- (-)	- (-)	- (-)	- (-)	10 (7,372,236)	0 (320,115,455)
**300,000,000**	**10,000,000**	- (-)	- (-)	- (-)	- (-)	- (-)	11 (4,696,280)

Table S9. Multi-criteria analysis of preventive and reactionary measures. The impact values are given on a scale from -5 (very unwanted impact) to +5 (very wanted impact) and in relation to not undertake any measure.

	**New culvert**		**Dry water pond**		**Macadam basin with permeable asphalt**		**Additional pump capacity**		**Expensive measures, e.g.** protective embankments, dikes/walls or even more complex constructions	
**Functionality**	Effect of intervention	Impact value	Effect of intervention	Effect of intervention	Effect of intervention	Impact value	Effect of intervention	Impact value	Effect of intervention	Impact value
Level of protection with regard to										
- direct impacts of both landslides and flooding: consequences for life and personal injuries, buildings, and infrastructure,<br>- indirect consequences of landslides, for example, potential flooding/energetic waves, meandering, and the related consequences for life, buildings, and infrastructure.	Complete protection under current projections.	5	Complete protection under current projections.	5	Complete protection with regard to current projections.	5	Require personnel to arrive to the site, distribute and start the pumps. Pumping also required the road to be closed after pumping is completed, resulting in the estimate of a closure of half the original time.	2	Complete protection under current projections.	5
Technical complexity of the intervention (short-term), and complexity of maintenance (long-term)	Require investment and time for construction but can be carried out without major shutdowns of traffic. Investment costs depend on if the measures are implemented into	-2	Require investment and time for construction but can be carried out without major shutdowns of traffic. Investment costs depend on if the measures are implemented into	-2	Require investment and time for construction but can be carried out without major shutdowns of traffic. Investment costs depend on if the measures are implemented into	-1	Require some additional storing and logistics, relatively low complexity.	0	Require large investment and long time for construction, as well as shutdowns of traffic.<br><br>More complex measures require	-5

	new construction or requiring retrofitting. If retrofitting is needed, socioeconomic costs due to reduced accessibility and likely lowered speeds during the installation are occurred.<br><br>Will also require maintenance to maintain its function.		new construction or requiring retrofitting. If retrofitting is needed, socioeconomic costs due to reduced accessibility and likely lowered speeds during the installation are occurred.<br><br>May also require some maintenance to maintain its function.		new construction or requiring retrofitting. If retrofitting is needed, socio-economic costs due to reduced accessibility and likely lowered speeds during the installation are occurred.<br><br>The macadam basin will not require maintenance, but the permeable asphalt may need to be cleared of clogged pores and more frequently rebuilt compared to normal asphalt.				relatively more maintenance and interventions.	
Environmental aspects										
- Global warming and energy consumption—impacts on emissions of greenhouse gases and carbon sequestration.	Requires energy and will give rise to carbon dioxide emissions during the culvert production (cement).	-2	Requires low amounts of energy and therefore has a low impact on carbon dioxide emissions.	0	CO_2 emissions and use of energy during constrction mainly due to transport of the heavy masses and some for blasting for material.	-2	Requires energy for pumping. The impact on global warming depends on the energy source. If the pumps are electrical the impact is low due to Sweden’s large share of renewable energy, if the pumps are diesel-based the impact will be large. In case of flooding event we assume diesel pumps being used.	-1	Requires a large amount of energy and material, likely concrete (cement) and steel, for the construction and will demand frequent in maintenance.	-5

- Air—emissions of toxic gases, emissions of particles, airborne bio-accumulative substances, emissions that contribute to eutrophication, acidification, oxidants, and formation of ground layer ozone.	Contribution is largely related to production and installation including redirection of traffic.	-1	Contribution is largely related to the installation.	0	Contribution is largely related to transport, production and installation.<br><br>Road dust binding is increased due to road and tyre wear from permeable asphalt (studded tyres should be avoided).	-2	Diesel pumps contribute to emissions while electrical pumps have low emission levels due to Sweden's large share of renewable energy. In case of flooding event we assume diesel pumps being used.	-1	Contribution is largely related to production and installation.<br><br>More complex alternatives emit relatively more pollutants.	-2
- Water quality, including quality of surface and groundwater (during construction in the short-term, and due to flood or landslide events in a long-term perspective)	Impact on water quality depends on how the excess water is handled. If sent to a treatment plant (which is not likely in the Swedish road system), but if released into the local environment, the risk of pollution increases. The installation is not expected to alter the spreading compared to no measure being taken.	0	Impact on water quality is positive due to reduced spreading of pollutants.	3	Impact on water quality depends on how the excess water is handled. If sent to a treatment plant, road and traffic-related pollutants are taken care of but if released into the local environment, the risk of pollution increases. The construction is not expected to alter the spreading compared to no measure being taken.	0	Impact on water quality depends on how the pumped water is handled. If sent to a treatment plant, road and traffic-related pollutants are taken care of but if released into the local environment, the risk of pollution increases. The measure is not expected to alter the spreading compared to no measure being taken.	0	A potential increased risks of contaminants in water during the construction.	-1
- Soil quality and terrestrial impacts, such as potential discharge of nutrients or contaminants from agricultural soil.	No or very small impact on soil quality.	0	Impact on soil quality is positive due to increased amount of vegetation and less spreading of pollutants.	2	No or very small impact on soil quality.	0	No or very small impact on soil quality.	0	Complex alternatives may require more soil, which can have some negative effect.	-1
- Ecosystem functions—fauna and flora (biodiversity,	Relatively small impacts except the effect of mining and raw material	0	Impact on ecosystem functions is positive due to the	2	Relatively small impacts except the effect mining and raw material	-1	No impact.	0	Complex alternatives demand more raw material and	-3

impacts on ecosystem functions and services such as fisheries, terrestrial, marine and limnological properties of high conservation value).	abstraction yield (removal of habitat areas), but the culvert also offers an improvent in fauna passage.		vegetative measure being favorable for biodiversity.		abstraction yield (removal of habitat areas).				take up more land and do therefore have larger impacts on habitat intrusions as well as larger barrier effects.	
- Flooding—time of inundation, piezometric level of aquifer.	100 percent protection, risk for flooding reduces to 0.	5	100 percent protection, risk for flooding reduces to 0.	5	100 percent protection, risk for flooding reduces to 0.	5	Protection can vary depending on the duration of the flood, from nearly 0 percent in short events to nearly 100 percent for long-term. If there are several events in the immediate area, efficiency can be reduced, due to the unavailability of pumps, transportation and personnel.	3	100 percent protection, risk for flooding reduces to 0.	5
Social aspects										
- Perception, such as concern or anxiety of flood risk/landslide risk, perception of other aspects of doing nothing or the intervention, such as aesthetics, attachment, perceived disturbances of construction, intervention, maintenance, and so on.	There are no local residents or activities that can be affected by the measure. It should therefore have marginal impact.	0	There are no local residents or activities that can be affected by the measure. It should therefore have marginal impact.	0	There are no local residents or activities that can be affected by the measure. It should therefore have marginal impact.	0	There are no local residents or activities that can be affected by the measure. It should therefore have marginal impact.	0	There are no local residents or activities that can be affected by the measures. They should therefore have marginal impacts.	0
- Socioeconomic aspects (not considered under	The measure contributes to counteract the	5	The measure contributes to counteract the	5	The measure contributes to counteract the	5	The measure contributes to counteract the	4	The measures contribute to counteract the	5

direct and indirect impacts) such as effects on the social fabric, jobs, business activity.	impact of socioeconomic costs related to flooding.		impact of socioeconomic costs related to flooding.		impact of socioeconomic costs related to flooding.		impact of socioeconomic costs related to flooding.		impact of socioeconomic costs related to flooding.	
- Health and recreation.	There are no local residents or activities that can be affected by the measure. It should therefore have marginal impact.	0	There are no local residents or activities that can be affected by the measure. It should therefore have marginal impact.	0	There are no local residents or activities that can be affected by the measure. It should therefore have marginal impact.	0	There are no local residents or activities that can be affected by the measure. It should therefore have marginal impact.	0	There are no local residents or activities that can be affected by the measures. They should therefore have marginal impacts.	0
Costs (see NPV results)										
Flexibility										
- High flexibility implies no-regret solutions, and reversibility of the system as well as potential to adapt to future needs such as more increase in extreme weather events than expected based on current climate change scenarios.	Large opportunity to change trajectory if climate change projections turn out to out to be erroneous.	5	Large opportunity to change trajectory if climate change projections turn out to out to be erroneous.	5	Large opportunity to change trajectory if climate change projections turn out to out to be erroneous.	5	Large opportunity to change trajectory if climate change projections turn out to out to be erroneous.	5	Very small opportunity to change trajectory if climate change projections turn out to out to be erroneous. .	-1 to -5 depending on type of construction
**Total**		13		25		13		11		-9 to -13 depending on type of construction

Table S10. Socioeconomic cost of flooding based on different values of AADT and queueing, SEK in 2017-year prices.

	Queueing				
**AADT**	0h	2.5h	5h	7.5h	10h
**75.0%**	1,417,582	3,042,082	8,403,929	15,089,344	21,789,838
**82.5%**	1,653,846	3,549,096	9,804,584	17,604,234	25,421,478
**100.0%**	1,890,110	4,056,110	11,205,239	20,119,125	29,053,118
**112.5%**	2,126,374	4,563,123	12,605,894	22,634,016	32,684,757
**125.0%**	2,362,637	5,070,137	14,006,549	25,148,906	36,316,397

Table S11. Net present value of a 1,000,000 SEK damage reduction due to preventive measure, based on economic life span and change factor.

	Change factor			
Economic life span (years)	0.90	0.95	1.00	1.05
10	5,861,894	7,623,998	10,000,000	13,206,787
20	7,905,810	12,188,767	20,000,000	34,719,252
30	8,618,480	14,921,863	30,000,000	69,760,790
40	8,866,972	16,558,269	40,000,000	126,839,763
50	8,953,616	17,538,045	50,000,000	219,815,396
60	8,983,827	18,124,674	60,000,000	371,262,904

Table S12. Change factor based on valuation change factor and discount rate.

	Discount rate			
Valuation change factor	0%	2%	3.5%	5%
-3%	0.970	0.951	0.937	0.924
-2%	0.980	0.961	0.947	0.933
-1%	0.990	0.971	0.957	0.943
0%	1.000	0.980	0.966	0.952
1%	1.010	0.990	0.976	0.962
2%	1.020	1.000	0.986	0.971
3%	1.030	1.010	0.995	0.981

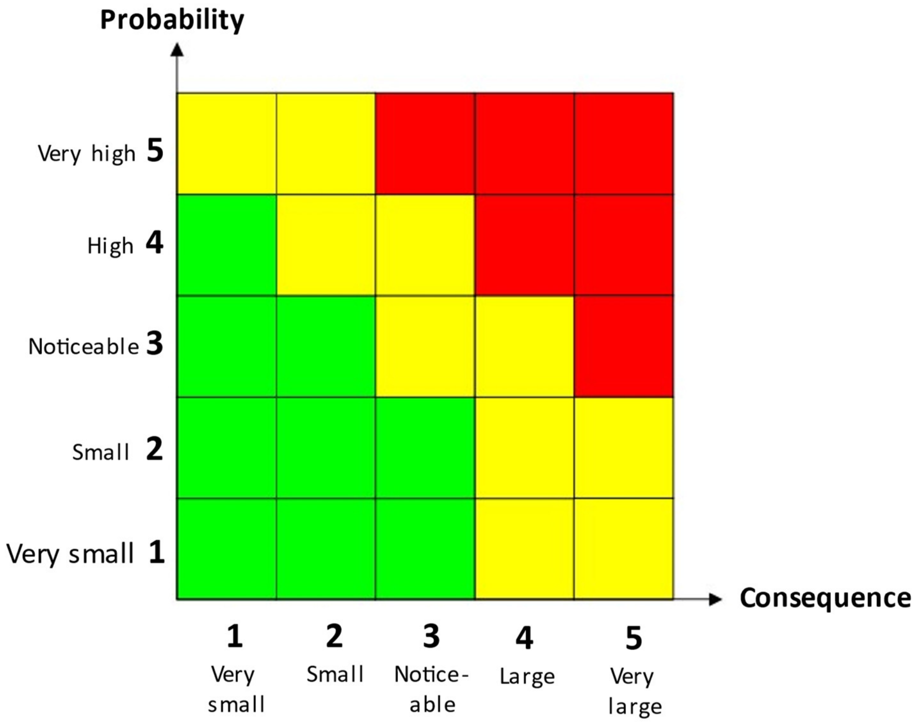

Risk class in the matrix:

- Class 3, high level of risk, usually not accepted
- Class 2, moderate level of risk, safety measures should be considered
- Class 1, low level of risk, usually accepted

Probability	Level	Once per (years)	= Safety factor
1	Very small	100 000 – 1 million	F > 1,5
2	Small	10 000 year – 100 000	1,4 < F ≤ 1,5
3	Noticeable	1 000 – 10 000	1,25 ≤ F ≤ 1,4
4	High	100 – 1000	1,1 ≤ F ≤ 1,25
5	Very high	10 – 100	F ≤ 1,1

Consequence	Level	Siffror
1	Very small	< 0,1 Mkr
2	Small	0,1-1 Mkr
3	Noticeable	1-10 Mkr
4	Large	10-100 Mkr
5	Very large	>100 Mkr

Figure S1. Example of risk matrix for natural disasters (flooding, landslide, and erosion) within STA. The figure is partly modified from Karlsson and Gunnarsson [60]. Green squares indicate acceptable risk, red squares indicate that the risk is not acceptable and yellow squares indicate that it is uncertain if acceptable or not and that further analyses are needed. At the bottom of the figure, there is information regarding intervals for, respectively, the probability class (to the left) and the consequence class (to the right).
